# MYB3R-mediated and cell cycle-dependent transcriptional regulation of a tobacco ortholog of *SCARECROW-LIKE28* in synchronized cultures of BY-2 cells

**DOI:** 10.5511/plantbiotechnology.23.0515a

**Published:** 2023-12-25

**Authors:** Keito Mineta, Junya Hirota, Kesuke Yamada, Takashi Itoh, Poyu Chen, Hidekazu Iwakawa, Hirotomo Takatsuka, Yuji Nomoto, Masaki Ito

**Affiliations:** 1 School of Biological Science and Technology, College of Science and Engineering, Kanazawa University, Kakuma-machi, Kanazawa, Ishikawa 920-1192, Japan; 2 Department of Biological Sciences, Graduate School of Science, The University of Tokyo, Hongo, Tokyo 113-0033, Japan

**Keywords:** Arabidopsis, cell cycle, tobacco BY-2 cells, transcriptional regulation

## Abstract

Although it is well known that hierarchical transcriptional networks are essential for various aspects of plant development and environmental response, little has been investigated about whether and how they also regulate the plant cell cycle. Recent studies on cell cycle regulation in *Arabidopsis thaliana* identified SCARECROW-LIKE28 (SCL28), a GRAS-type transcription factor, that constitutes a hierarchical transcriptional pathway comprised of MYB3R, SCL28 and SIAMESE-RELATED (SMR). In this pathway, MYB3R family proteins regulate the G2/M-specific transcription of the *SCL28* gene, of which products, in turn, positively regulate the transcription of *SMR* genes encoding a group of plant-specific inhibitor proteins of cyclin-dependent kinases. However, this pathway with a role in cell cycle inhibition is solely demonstrated in *A. thaliana*, thus leaving open the question of whether and to what extent this pathway is evolutionarily conserved in plants. In this study, we conducted differential display RT-PCR on synchronized *Nicotiana tabacum* (tobacco) BY-2 cells and identified several M-phase-specific cDNA clones, one of which turned out to be a tobacco ortholog of *SCL28* and was designated *NtSCL28*. We showed that *NtSCL28* is expressed specifically during G2/M and early G1 in the synchronized cultures of BY-2 cells. *NtSCL28* contains MYB3R-binding promoter elements, so-called mitosis-specific activator elements, and is upregulated by a hyperactive form of NtmybA2, one of the MYB3R proteins from tobacco. Our study indicated that a part of the hierarchical pathway identified in *A. thaliana* is equally operating in tobacco cells, suggesting the conservation of this pathway across different families in evolution of angiosperm.

For proper progression through and exit from cell cycle, it is crucial to regulate the transcription of genes with cell cycle-related functions. There are two main groups of cell cycle-regulated genes with different expression profile: G1/S-specific and G2/M-specific genes (Berckmans and De Veylder 2009). While the G1/S-specific genes often encode proteins with various roles necessary for DNA replication, products of G2/M-specific genes typically play roles in initiating and progressing through mitosis and cytokinesis (Berckmans and De Veylder 2009). Due to the sequential mRNA expression of the G1/S- and G2/M-specific genes, cells are believed to be able to control the proper order of various events occurring in the cell cycle. For the cell cycle-regulated transcription, two main families of transcription factors, E2F and MYB3R, play essential roles. E2F, interacting with Dimerization Partner (DP), regulates the transcription of G1/S genes by binding to E2F motifs in their promoter regions, whereas MYB3R binds to mitosis-specific activator (MSA) elements to control the transcription of G2/M-specific genes (Berckmans and De Veylder 2009; Magyar et al. 2016). It was reported that E2F/DP and MYB3R are present in a common large multi-protein complex, called DREAM complex, which plays cell cycle-related and -unrelated functions (Kobayashi et al. 2015; Lang et al. 2021; Ning et al. 2020).

Various biological processes are governed by a hierarchical transcription network, in which master regulators directly regulate other transcription factors, further regulating downstream target genes. Notably, studies in yeasts indicated that cell cycle transcriptional activators functioning at one stage of the cell cycle regulate transcriptional activators functioning at the next stage (Simon et al. 2001). This serial regulation of transcriptional activators forms a connected regulatory network that is a cycle itself. In human cells, a cell cycle transcription factor, FoxM1, is directly regulated by E2F and functions as a phase-specific component of DREAM-related complexes for activating many downstream G2/M-specific genes (Millour et al. 2011). However, in plants, there is limited knowledge about the hierarchical network composed of cell cycle transcription factors, such as E2F and MYB3R.

A GRAS-type transcription factor, *SCARECROW-LIKE28* (*SCL28*), has recently been identified as a direct target gene regulated by MYB3R in *Arabidopsis thaliana* (Arabidopsis). Under the control of MYB3R, the *SCL28* mRNA is specifically expressed during G2 and M phases, just like *CYCB1;1* and *CYCB1;2* encoding mitotic cyclins, as observed in root meristems and cell cultures of Arabidopsis (Goldy et al. 2021; Nomoto et al. 2022). SCL28 inhibits the cell cycle progression at G2 by activating the transcription of genes encoding plant-specific CDK inhibitors of the SIAMESE-RELATED (SMR) family. Seven members out of 17 were identified as direct downstream targets of SCL28. Therefore, these studies uncovered the presence of a plant-specific hierarchical transcriptional cascade, MYB3R-SCL28-SMR, as a mechanism for negatively regulating the cell cycle, thereby controlling proper cell size (Goldy et al. 2023; Nomoto et al. 2022; Yamada et al. 2022). Although SCL28 orthologs have been studied in some plant species, such as rice (Hirano et al. 2017), this transcriptional hierarchy has not been investigated in plants except Arabidopsis, making it unclear whether this cascade is a general regulatory mechanism conserved in plants.

An *SCL28* ortholog in *Nicotiana tabacum* (tobacco) was identified by our screening aimed at isolating phase-specific cDNAs from tobacco BY-2 cells using a differential display RT-PCR method. The synchronization of BY-2 cells was induced by treating 7-day-old cells with aphidicolin (5 mg l^−1^), an inhibitor of DNA polymerase α, for 24 h and the subsequent removal of the drug (Kumagai-Sano et al. 2006). We extracted total RNA from cells at 0 and 7 h after the removal of aphidicolin, corresponding to S and M phases in the cell cycle, respectively, and used them for synthesizing first-strand cDNA with a method previously described (Yoshida et al. 2001). Next, we conducted a differential display RT-PCR using primers of arbitrary sequences (Yoshida et al. 2001) and identified DNA fragments specific to either the S or M phase in the cell cycle. They were subsequently cloned in the pGEM-T Easy vector (Promega) and sequenced. One of the M phase-specific cDNAs, *E1M* (clone number E1 obtained from M phase-specific cDNAs), encoded a partial amino acid sequence displaying similarities with a part of the GRAS family transcription factors. We could determine a nucleotide sequence containing an entire open reading frame of 2,028 bp, encoding a putative protein comprised of 675 amino acids, by conducting a 3′ and 5′ rapid amplification of cDNA ends (RACE) with a FirstChoice RLM-RACE Kit (Thermo Fisher Scientific). When entire protein sequences were compared, those encoded by *E1M* closely resembled the Arabidopsis SCL28 (hereafter called AtSCL28 to distinguish from homologs in other plant species) with an identity score of 53%. A higher identity score (65%) was found when the comparison was made within the conserved GRAS domain that is located in C-terminal half of the proteins (Figure 1A). The other GRAS proteins from Arabidopsis also displayed a sequence similarity to E1M within the GRAS domain, whereas a negligible similarity was found outside of the GRAS domain, except for AtSCL28, which still displayed 38% of identity in N-terminal halves of the proteins (Figure 1B). Phylogenetic analyses of GRAS proteins from Arabidopsis and tobacco indicated that E1M represents a tobacco ortholog of AtSCL28 (Figure 1C). Therefore, E1M will hereafter be called NtSCL28.

**Figure 1:**
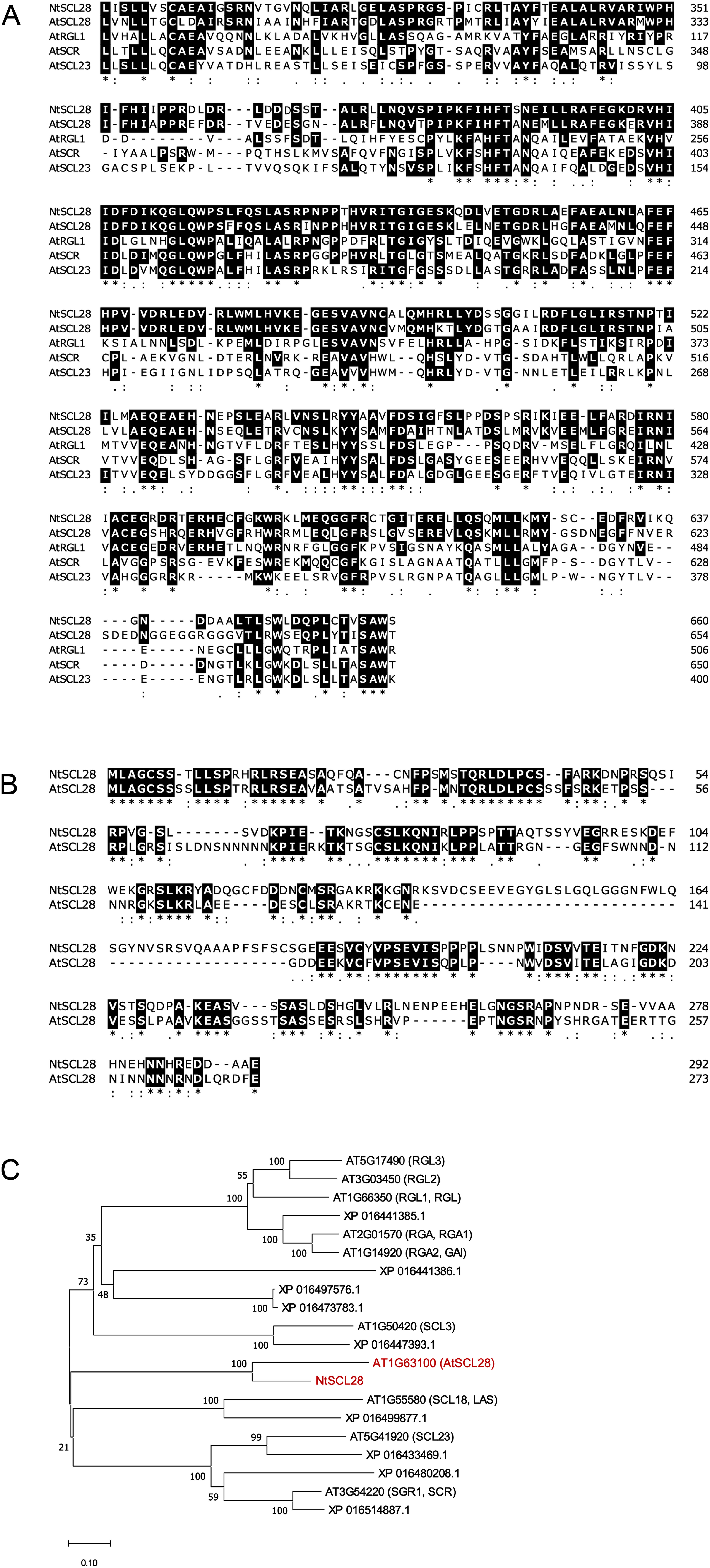
Figure 1. NtSCL28 from tobacco is orthologous to Arabidopsis AtSCL28. (A) Comparison of amino acid sequences within the GRAS domain among NtSCL28, AtSCL28, and other Arabidopsis GRAS proteins (AtRGL1, AtSCR, and AtSCL23) that are highly similar to AtSCL28. The identical amino acids are indicated in white letters on a black background. Below the protein sequences, residues identical across all five sequences are indicated by asterisks (*), conservative substitutions by colons (:), and semi-conserved substitutions by periods (.). (B) Comparison of amino acid sequences outside the GRAS domain between NtSCL28 and AtSCL28. Below the protein sequences, residues are marked as in (A). (C) Phylogenetic tree of GRAS proteins from Arabidopsis and tobacco. Protein names beginning with “AT” are from Arabidopsis, and those beginning with “XP” are from tobacco. A BLAST search was conducted using the amino acid sequence of AtSCL28 as the query, and the top 10 proteins with the highest similarity score were collected from Arabidopsis and tobacco. The phylogenetic tree was generated with MEGA7 using the maximum likelihood method and Jones–Taylor–Thornton matrix-based model with 1,000 bootstrap replicates.

To analyze expression patterns of *NtSCL28* in the cell cycle, BY-2 cells were synchronized by aphidicolin treatment as described above. After release from the aphidicolin block, the progression through the cell cycle was monitored by analyzing time-course changes in the mitotic index (Figure 2A). Total RNA was extracted from cells harvested every 60 min after the removal of aphidicolin using TRIzol RNA Isolation Reagents (Thermo Fisher Scientific), which was further used for cDNA synthesis and quantitative reverse transcription-PCR (qRT-PCR). cDNA was synthesized using ReverTra Ace qPCR RT Kit (TOYOBO), and qPCR was performed using THUNDERBIRD Next SYBR qPCR Mix (TOYOBO) on StepOnePlus Real-Time PCR System (Applied Biosystems). qRT-PCR was conducted using primer pairs specific to *NtSCL28* and *NtSMOS1* (Supplementary Table S1). *NtSCL28* mRNA levels increased at 6 h and reached a maximum at 9 h after release from the aphidicolin block (Figure 2B). After that, the mRNA levels decreased and reached close to the basal levels at 14 h. Combined with temporal changes of the mitotic index with a clear peak occurring at 7 h, we concluded that the *NtSCL28* mRNA is preferentially accumulated in cells during the late G2, M, and early G1 phases of the cell cycle.

**Figure 2:**
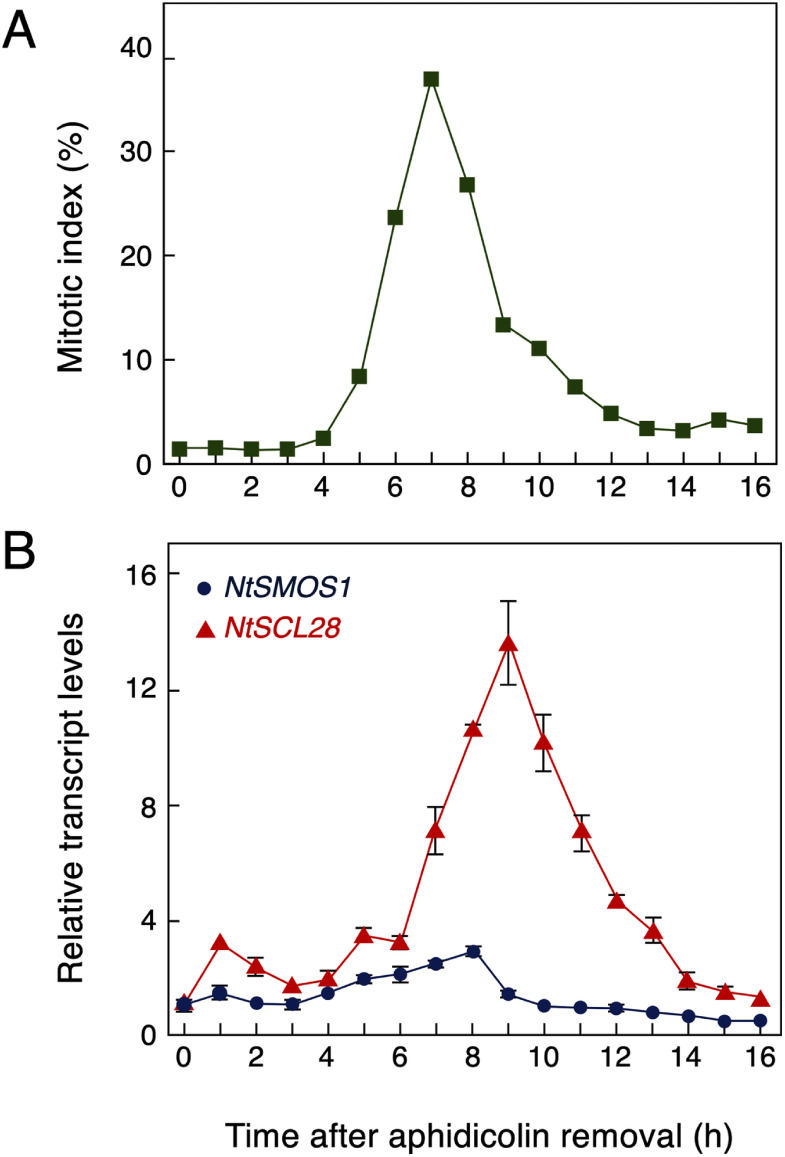
Figure 2. Accumulation patterns of mRNAs for *NtSCL28* and *NtSMOS1* in synchronized BY-2 cells. (A) Change in the mitotic index during synchronized cultures of BY-2 cells. (B) qRT-PCR analysis of *NtSCL28* and *NtSMOS1* mRNAs during synchronized cultures of BY-2 cells. For qRT-PCR analysis, RNA samples obtained in our previous report were used (Kato et al. 2009). Expression levels of each transcript were normalized by the levels of *EF1*α expression. They are expressed as relative values, with transcript levels at 0 h set to 1.0. Error bars represent standard deviation (SD) for *n*=3.

We previously demonstrated that AtSCL28 interacts with an AP2-type transcription factor, AtSMOS1, and forms an active heterodimer with an ability to bind specific target motifs (Nomoto et al. 2022). Studies in rice also indicated that SMOS2, the rice ortholog of AtSCL28, had a protein–protein interaction with SMOS1, the rice ortholog of AtSMOS1 (Hirano et al. 2017). By conducting a BLAST search against the *Nicotiana tabacum* proteome using the protein sequence of AtSMOS1 as the query, we found a tobacco protein, XP_016477494, showing a close similarity with AtSMOS1 and rice SMOS1 (Supplementary Figure S1). Phylogenetic analyses of these AP2-type transcription factors suggested that XP_016477494 is orthologous to AtSMOS1 and SMOS1 and was thus designated NtSMOS1. The 3D protein structures predicted by AlphaFold2 (https://colab.research.google.com/github/sokrypton/ColabFold/blob/main/AlphaFold2.ipynb (Accessed Apr 17, 2023)) showed closely similar structures for both SMOS1 and SCL28 when compared between tobacco and Arabidopsis, supporting the idea that two tobacco proteins, NtSCL28 and NtSMOS1, may also interact with each other as was shown in Arabidopsis (Supplementary Figure S2). The accumulation pattern of *NtSMOS1* mRNA was similarly examined by qRT-PCR using the same total RNA samples from synchronized BY-2 cells. Unlike *NtSCL28* mRNA that displayed a dramatic expression change in the cell cycle, only a moderate change of *NtSMOS1* mRNA levels was observed with a peak expression at 8 h, which was much less pronounced in comparison with that of *AtSCL28* mRNA (Figure 2B). This observation was consistent with our previous results indicating a uniform, instead of patchy, expression of *AtSMOS1-GFP* driven by its own promoter in root meristems of transgenic Arabidopsis (Nomoto et al. 2022). Therefore, the observed expression of *NtSCL28* and *NtSMOS1* was generally consistent with our report showing cell cycle-dependent and -independent expression of *AtSCL28* and *AtSMOS1*, respectively, in Arabidopsis (Nomoto et al. 2022).

The observed G2/M-specific transcript accumulation suggests that *NtSCL28* is expressed in actively proliferating cells and functions exclusively in cells undergoing the cell cycle progression. To examine whether the expression of *NtSCL28* and *NtSMOS1* is specific to proliferating cells or persists after the exit from cell proliferation, we utilized asynchronous cultures of BY-2 cells. After subculturing, rapid cell proliferation began by day 1 and continued until day 4, resulting in an exponential increase in cell numbers. After that, the increase in cell number gradually slowed and ceased on day 7, representing entry into the stationary phase (Figure 3A). Cells harvested every 24 h after subculturing were used for RNA extraction and subsequent qRT-PCR analyses. *NtSCL28* transcript levels were negligible at day 0, then rapidly increased by day 1 and stayed high for days 1–6 after subculturing. Thereafter, the levels decreased and remained very low during days 7–9, corresponding to the stationary phase (Figure 3B). In contrast, *NtSMOS1* transcript levels did not dramatically change throughout the culture period, suggesting that *NtSMOS1* is expressed in both cells during and after exit from proliferation. These results in asynchronous cultures suggest that NtSCL28 may fulfil its function exclusively in proliferating cells by forming a heterodimer with NtSMOS1 and may not have a direct role in quiescent cells that have already exited proliferation and do not express *NtSCL28*. *NtSMOS1*, also expressed in quiescent cells, may have an additional role independent of NtSCL28, as suggested for AtSMOS1 in Arabidopsis (Nomoto et al. 2022).

**Figure 3:**
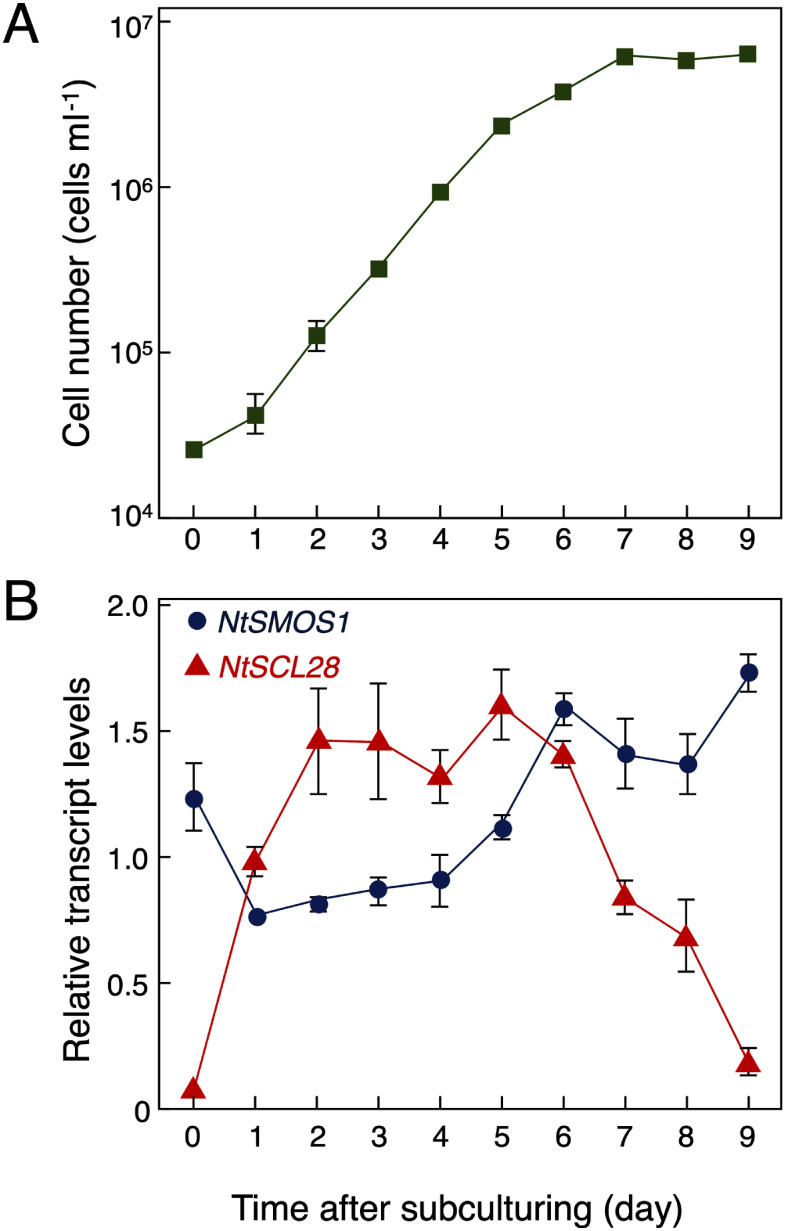
Figure 3. Accumulation of *NtSCL28* and *NtSMOS1* mRNAs in proliferating and quiescent BY-2 cells (A) Increase in cell numbers after subculturing. For subculturing, 7-day-old BY-2 cells were diluted 100 times with fresh medium and cultured for up to 9 days. (B) Temporal changes in transcript accumulation in BY-2 cells after subculturing. For qRT-PCR analysis, RNA samples obtained in our previous report were used (Takatsuka et al. 2021). Expression levels of each transcript were normalized by the levels of *EF1*α expression and are expressed as relative values, with the average transcript level over all time points set to 1.0.

The G2/M-specific transcription of *AtSCL28* is regulated by MYB3R transcription factors that bind to MSA elements in Arabidopsis. To understand the transcriptional regulation of *NtSCL28*, we examined if *NtSCL28* also contains MSA elements in its upstream region. Thermal asymmetric interlaced PCR (TAIL-PCR) was conducted using genomic DNA from BY-2 cells to isolate genomic fragments corresponding to the *NtSCL28* promoter. The TAIL-PCR procedure was carried out as described previously (Liu et al. 1995), using arbitrary degenerate primers and primers specific to *NtSCL28*. One amplified fragment contained a nucleotide sequence overlapping with the *NtSCL28* cDNA, confirming that the genomic regions of *NtSCL28* were successfully isolated. In this experiment, we could determine a 3.7 kb sequence upstream of the predicted start codon of *NtSCL28*. Based on our result of 5′ RACE described above, we could map the transcription start site (TSS) of *NtSCL28*, which is located 921 bp upstream of the predicted start codon. By investigating the nucleotide sequence of the upstream region, we could recognize typical MSA-like sequences repeated twice in a forward orientation (AACGG in sense strand) at the positions located just upstream of TSS (−220 and −191) (Figure 4A). Likewise, our previous study showed that *AtSCL28* contains four MSA-like motifs, among which three are present in a forward orientation at the positions −34, −73 and −101, whereas the other one exists in a reverse orientation (CCGTT in sense strand) at the position −54 (Nomoto et al. 2022). Our present and previous observations confirmed the view that MSA elements most frequently exist in proximity to the TSS, and that number and orientation of the motifs are not generally fixed, instead rather variable among genes regulated by MYB3R (Haga et al. 2011; Kato et al. 2009).

**Figure 4:**
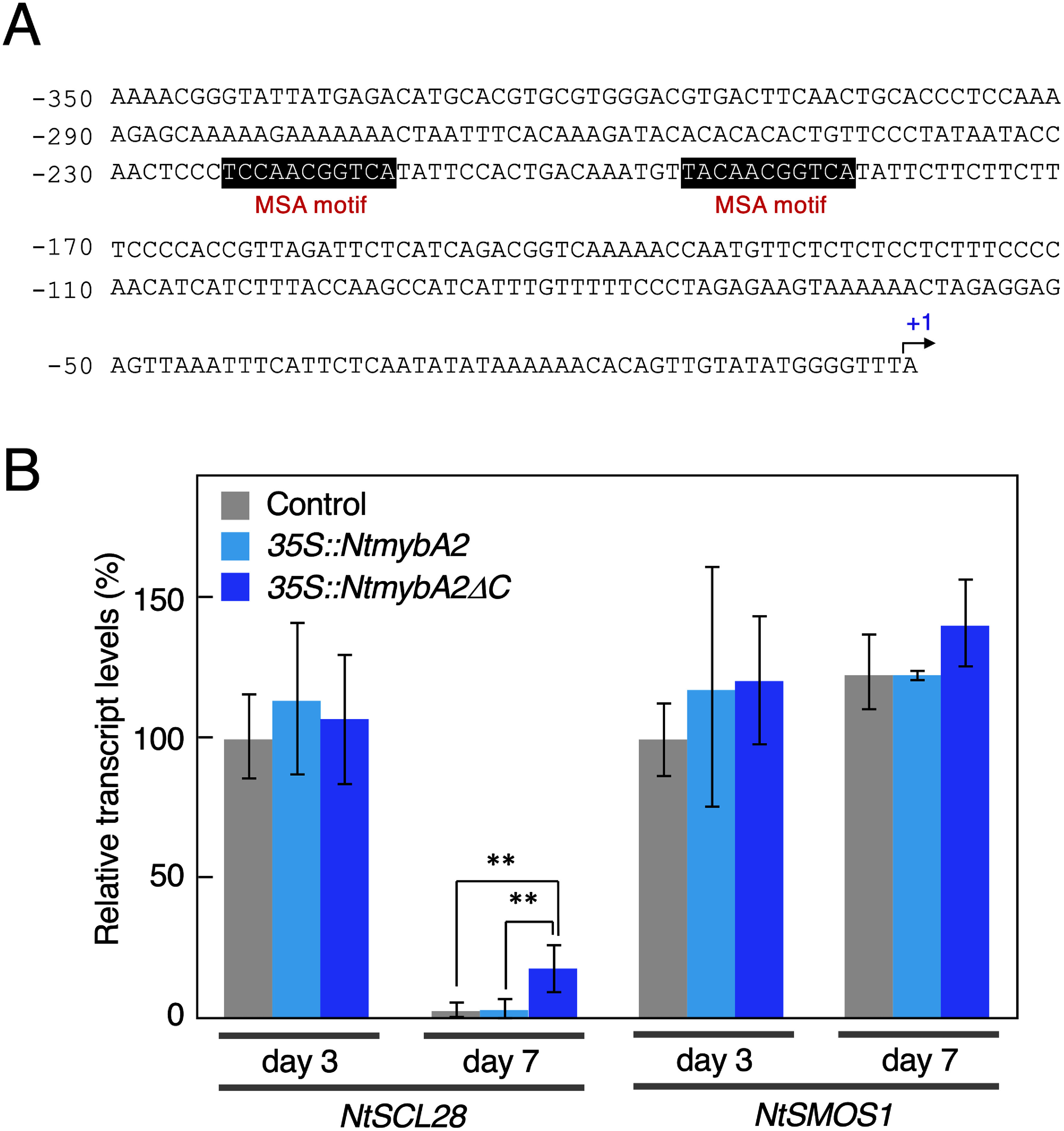
Figure 4. *NtSCL28*, but not *NtSMOS1*, is regulated by NtmybA2. (A) Nucleotide sequence of promoter region from *NtSCL28*. The genomic fragment of *NtSCL28* was obtained by TAIL-PCR and sequenced. Two consensus MSA motifs near the TSS are indicated in white letters on a black background. TSS is shown by +1. (B) Upregulation of *NtSCL28* in transgenic BY-2 cells carrying *35S::NtmybA2ΔC*. Tobacco BY-2 cells were transformed with the vector alone (control), *35S::NtmybA2*, or *35S::NtmybA2ΔC* by the *Agrobacterium*-mediated method. For each construct, total RNA was extracted from cells on day 3 (logarithmic phase) and day 7 (stationary phase) after subculturing. For qRT-PCR analysis of *NtSCL28* and *NtSMOS1*, RNA samples obtained in our previous report were used (Kato et al. 2009). The results of the qRT-PCR analysis were normalized to the expression of *EF1*α mRNAs and are expressed as relative values with levels of transcripts in control cells at day 3 set to 1.0. Error bars represent SD for *n*=3. ** indicates significance at *p*<0.01 (two-sided Student’s *t*-test).

In tobacco, two MYB3R proteins—NtmybA1 and NtmybA2—with a function for transcriptional activation have been reported (Ito et al. 2001). NtmybA2 contains a negative regulation domain in its C-terminal region, a deletion of which resulted in a significant enhancement of the transactivating activity of NtmybA2 (Kato et al. 2009). To examine whether MYB3Rs regulate *NtSCL28* transcription, we created transgenic BY-2 cells overexpressing *NtmybA2* with a C-terminal truncation (*NtmybA2ΔC*), which acts as a constitutively active form of NtmybA2 (Kato et al. 2009). *35S::NtmybA2ΔC* constructs were introduced into BY-2 cells by *Agrobacterium*-mediated gene transfer to establish stable transgenic lines. The resulting transgenic lines were then used for analyzing *NtSCL28* mRNA levels by qRT-PCR as we previously reported (Kato et al. 2009). When 7-day-old cells in the stationary phase were analyzed, we observed a significant upregulation of *NtSCL28* mRNA in *35S::NtmybA2ΔC* cells, about seven times compared with control cells carrying an empty vector (Figure 4B). In contrast, such upregulation of *NtSCL28* was not observed in *35S::NtmybA2* cells expressing full-length *NtmybA2*. The upregulation of *NtSCL28* was specific to cells in the stationary phase, as we observed mRNA levels of *NtSCL28* unchanged in 3-day-old cells of both *35S::NtmybA2ΔC* and *35S::NtmybA2* lines (Figure 4B). Similar expression changes had been observed for *CYCB1;3*, a canonical target of NtmybA2 in our previous study (Kato et al. 2009). The upregulation of these G2/M-specific genes is not due to delayed entry into stationary phase, because we previously reported that *35S::NtmybA2ΔC* and control transgenic BY-2 cells showed equivalent downregulation of the G1/S genes (*PCNA* and *CYCA3;1*) at day 7, whose expression is dependent on cell proliferation, but is not regulated by NtmybA2 (Kato et al. 2009). Our results, therefore, suggest that *NtSCL28* is a direct target gene transcriptionally activated by NtmybA2.

Finally, we analyzed the activity of the *NtSCL28* promoter during the cell cycle in BY-2 cells. The promoter fragment up to −2,202 was used to create a fusion construct with the luciferase (LUC) reporter gene in the pBI-LUC binary vector (Ito et al. 1998), and the resulting *proNtSCL28::LUC* construct was introduced into BY-2 cells by *Agrobacterium*-mediated gene transfer. Plasmid construction and transformation of BY-2 cells were conducted as described previously (Kato et al. 2009). Synchronized cultures were induced using the transgenic lines by aphidicolin treatment, and cells were harvested every 60 min after release from the aphidicolin block for measuring LUC activity. LUC activity was analyzed using a PicaGene Luminescence Kit (Fujifilm) on a luminometer (Lumat LB9501, Berthold Japan) as described previously (Ito et al. 1998). The time-course change in mitotic index indicated that cells underwent mitosis at 5–9 h with a peak level at 6 h after aphidicolin removal (Figure 5A). LUC activity stayed low during the first 4 h after release from aphidicolin block, followed by a rapid increase of LUC activity and reached a plateau at 9 h (Figure 5A). Because the rapid increase of LUC activity coincided with the increase of the mitotic index, we concluded that the *NtSCL28* promoter could direct the G2/M-specific transcription of the downstream *LUC* gene (Ito et al. 1998). We also used promoter regions from the Arabidopsis *AtSCL28* gene to examine cell cycle-dependent reporter expression by creating *proAtSCL28::LUC* constructs. By introducing this construct into BY-2 cells, we found that LUC activity changed in synchronized cultures with a similar pattern to that observed in *proNtSCL28::LUC* cells (Figure 5B). This result suggests that *AtSCL28* and its tobacco ortholog *NtSCL28* are transcriptionally regulated by a common mechanism, which probably involves MYB3R transcription factors binding to the MSA elements in their promoters.

**Figure 5:**
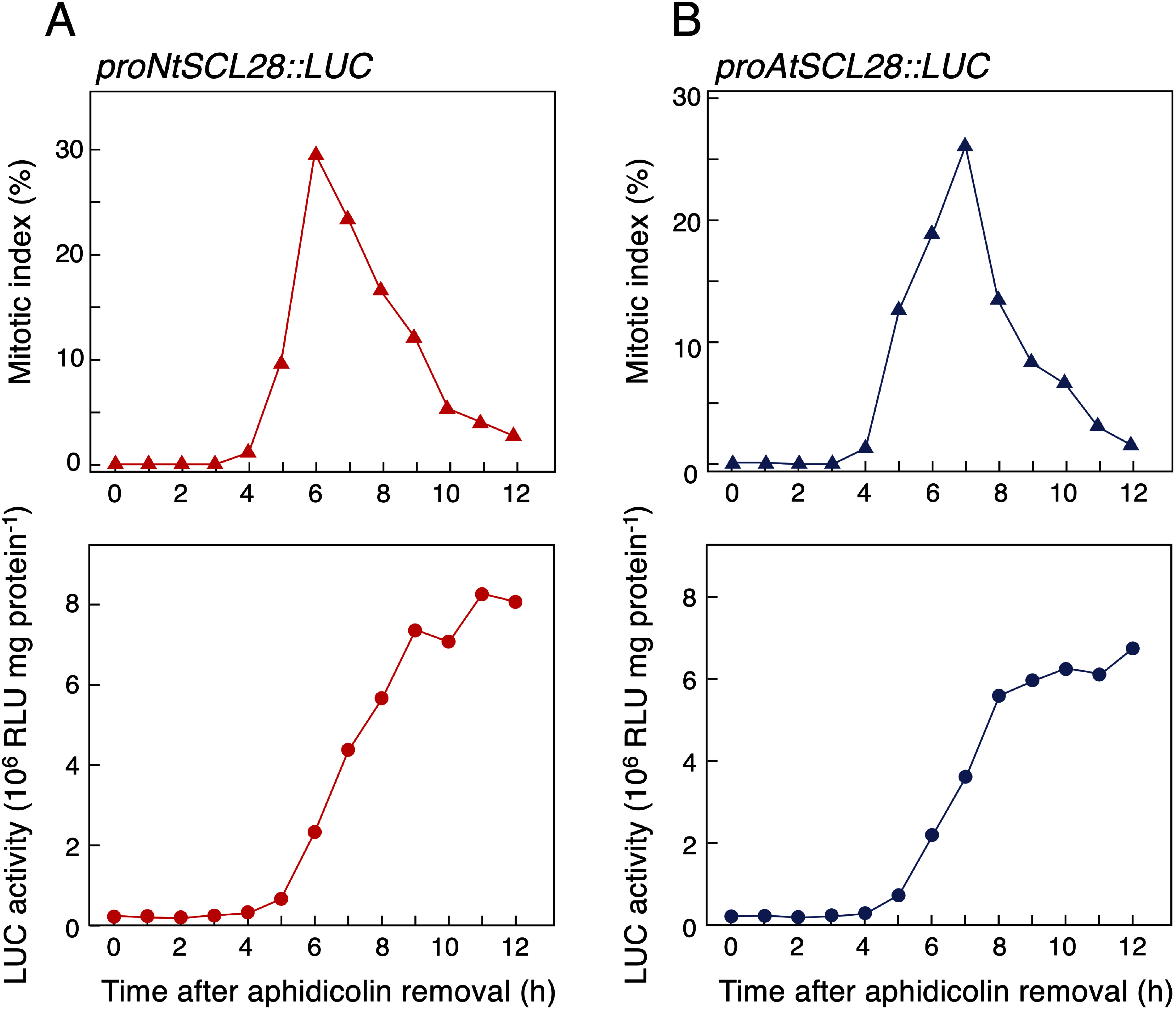
Figure 5. Cell cycle-dependent promoter activities of *NtSCL28* and *AtSCL28*. (A) Changes of *NtSCL28* promoter activity during synchronized cultures of BY-2 cells. Transgenic BY-2 cells carrying *proNtSCL28::LUC* construct were synchronized by aphidicolin treatment, and time-course changes of mitotic index (upper panel) and LUC activities (lower panel) were analyzed after release from aphidicolin block. (B) Changes of *AtSCL28* promoter activity during synchronized cultures of BY-2 cells. Transgenic BY-2 cells carrying the *proAtSCL28::LUC* construct were analyzed as in (A). RLU, relative light unit.

In conclusion, we identified NtSCL28 and NtSMOS1, tobacco orthologs of Arabidopsis AtSCL28 and its dimerization partner AtSMOS1, respectively. In BY-2 cells, *NtSCL28* is expressed in a cell cycle-dependent manner and transcriptionally activated by constitutive active form of NtmybA2, a tobacco MYB3R activator, whereas *NtSMOS1* is not activated by NtmybA2 and less clearly regulated during the cell cycle. Consistently, *NtSCL28* expression is specific to proliferating cells, unlike *NtSMOS1*, which is expressed in both proliferating and quiescent BY-2 cells. Our results indicate that a transcriptional regulatory pathway, MYB3R-SCL28 is operating not only in Arabidopsis but also in tobacco cells, suggesting an evolutionary conservation of this pathway, at least across different families in flowering plants. Our idea on this evolutionary conservation was also strongly supported by our heterologous experimental systems, in which the Arabidopsis *SCL28* promoter is correctly regulated during the cell cycle in tobacco cells.

## Abbreviations

Arabidopsis: *Arabidopsis thaliana*
DP: Dimerization Partner
LUC: luciferase
MSA: mitosis-specific activator
TAIL-PCR: thermal asymmetric interlaced PCR
TSS: transcription start site

## Accession numbers

Nucleotide sequence data reported in this study are available in the DDBJ/EMBL/GenBank database under the following accession numbers: LC726428 (*NtSCL28* mRNA), LC726429 (*NtSCL28* gene).

## Description of Supplementary Files

Supplementary Figure S1. Tobacco ortholog of AtSMOS1

Supplementary Figure S2. AlphaFold predictions of 3D protein structures of SMOS1 and SCL28

Supplementary Table S1. Primer list
