# Supplementary material for: MYB3R-mediated and cell cycle-dependent transcriptional regulation of a tobacco ortholog of SCARECROW-LIKE28 in synchronized cultures of BY-2 cells
Source: Plant Biotechnol (Tokyo). 2023 Dec 25;40(4):353–9. doi: 10.5511/plantbiotechnology.23.0515a (PMC10905365; doi:10.5511/plantbiotechnology.23.0515a)
Supplement: Supplementary Data [file plantbiotechnology-40-4-23.0515a-s001.pdf]

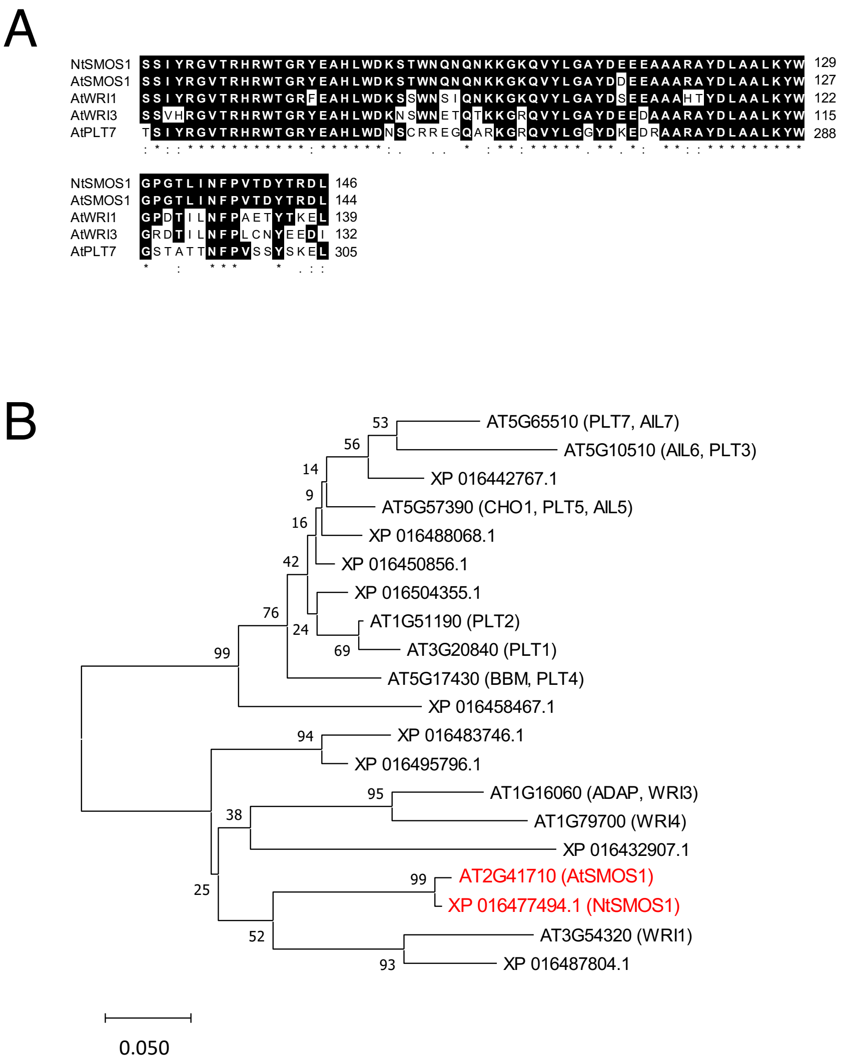

Supplementary Figure S1. Tobacco ortholog of AtSMOS1. (A) Comparison of amino acid sequences within the AP2 domain among NtSMOS1, AtSMOS1, and other Arabidopsis AP2-type proteins (AtWRI1, AtWRI3, and AtPLT7) that are highly similar to AtSMOS1. The identical amino acids are shown in white letters on a black background. Below the protein sequences, residues identical across all five sequences are indicated by asterisks (*), conservative substitutions by colons (:), and semi-conserved substitutions by periods (.). Amino acid position numbers are indicated on the left of the sequences. (B) Phylogenetic tree of AP2-type proteins from Arabidopsis and tobacco. Protein names beginning with “AT” are from Arabidopsis, and those beginning with “XP” are from tobacco. A BLAST search was conducted using the amino acid sequence of AtSMOS1 as a query, and the top 10 proteins with the highest similarity score were collected from Arabidopsis and tobacco. The phylogenetic tree was generated with MEGA7 using the maximum likelihood method and Jones–Taylor–Thornton matrix-based model with 1,000 bootstrap replicates.

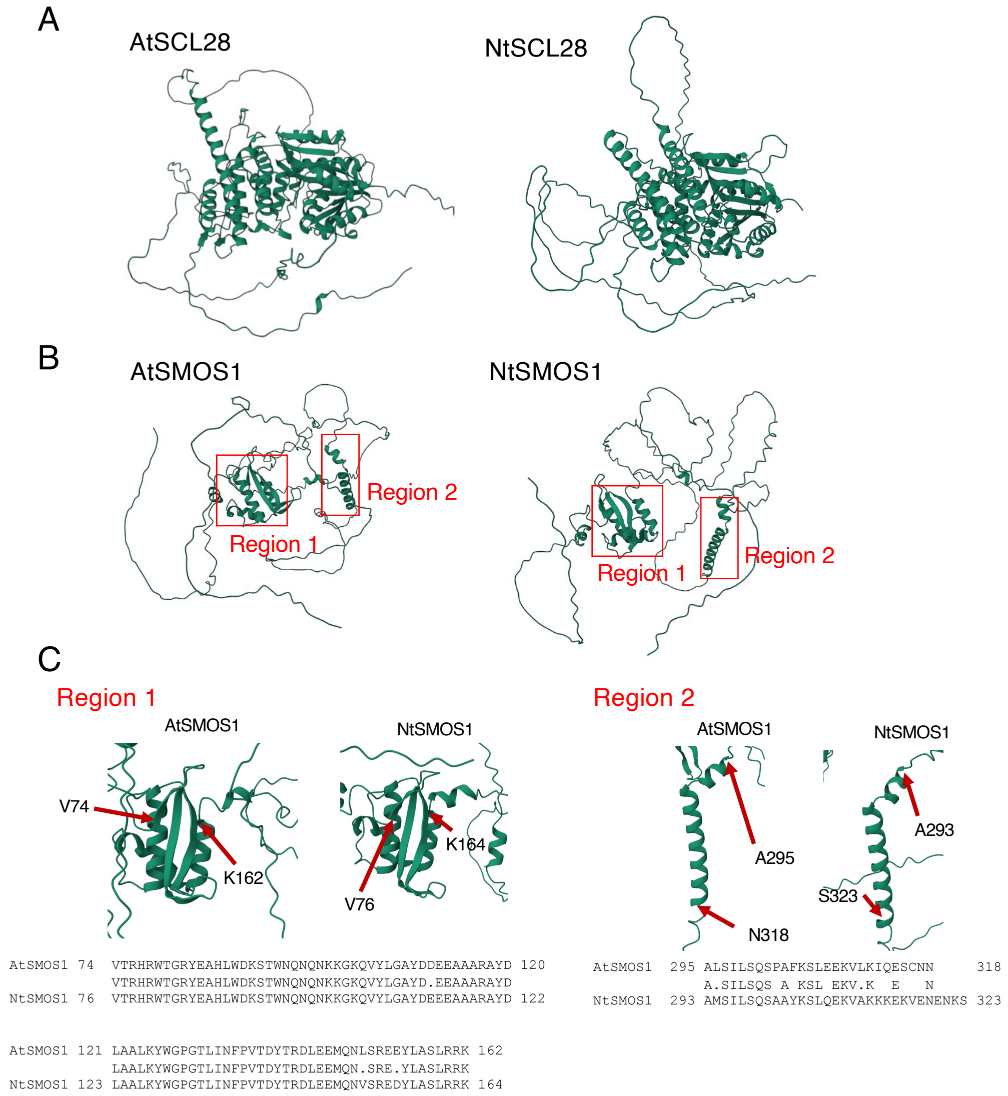

Supplementary Figure S2. Predicted protein structures of SCL28 and SMOS1 from Arabidopsis and tobacco. Protein structures were predicted by AlphaFold2 using complete amino acid sequences of corresponding proteins.

(A) Predicted 3D structures of AtSCL28 and NtSCL28.

(B) Predicted 3D structures of AtSMOS1 and NtSMOS1. Two main predicted structural domains were found at similar regions (Region 1 and Region 2) of each protein.

(C) Expanded view of predicted structural domains corresponding to Region 1 and Region 2 of AtSMOS1 and NtSMOS1. Amino acid sequence alignments of these regions are shown for comparing AtSMOS1 and NtSMOS1.

To predict protein structures, AlphaFold2 was used through a google colaboratory, ColabFold v1.5.2 (https://colab.research.google.com/github/sokrypton/ColabFold/blob/main/AlphaFold2.ipynb) following the instructions with default parameter (Jumper et al. 2021). AlphaFold2 produced PDB files were processed using Mol* 3D Viewer (https://www.rcsb.org/3d-view) to visualize protein structures (Sehnal et al. 2021).

Jumper J, Evans R, Pritzel A, Green T, Figurnov M, Ronneberger O, Tunyasuvunakool K, Bates R, Žídek A, Potapenko A, Bridgland A, Meyer C, Kohl SAA, Ballard AJ, Cowie A, Romera-Paredes B, Nikolov S, Jain R, Adler J, Back T, Petersen S, Reiman D, Clancy E, Zielinski M, Steinegger M, Pacholska M, Berghammer T, Bodenstein S, Silver D, Vinyals O, Senior AW, Kavukcuoglu K, Kohli P, Hassabis D. (2021) Highly accurate protein structure prediction with AlphaFold. *Nature* 596 : 583-589

Sehnal D, Bittrich S, Deshpande M, Svobodová R, Berka K, Bazgier V, Velankar S, Burley SK, Koča J, Rose AS. (2021) Mol* Viewer: modern web app for 3D visualization and analysis of large biomolecular structures. *Nucleic Acids Res* 49: W431-W437

Supplementary Table S1 Primer list

Primer name	Orientation	Sequence (5' - 3')	Purpose of PCR
E1M-Q5	Fwd	AATGTCACTGGTGTTAATCAAC	qRT-PCR for NtSCL28
E1M-S2	Rev	GATGTGAGGCCAAATCCTTGCAAC	
EF1a-F1	Fwd	GTAGGATACAACCCTGACAAGATC	qRT-PCR for E1Fα
EF1a-Q3	Rev	TCATTAATCTGGTCAAGAGCATC	
NtSMOS1-Q1	Fwd	AACGTAGATCCCTTAACAGAC	qRT-PCR for NtSMOS1
NtSMOS1-Q2	Rev	TAATCTGAAGTTGCTTCGCTC	
E1Mp5	Fwd	AACTGCAGTGTGTCAACAAGCACATGCAACGA	Construcrtion of proNtSCL28::LUC
E1Mp3-1	Rev	AAGTCGACAAGCCCCACTACCCAAAAAAGCTA	
AtE1Mp5	Fwd	AACTGCAGTTTGCCCATCTTTATTAATAGGCT	Construcrtion of proAtSCL28::LUC
AtE1Mp3-1	Rev	AAGTCGACAACCCAATTCAAGAGATGGTCTAC	
